# Abdominal pain in adolescent females: a single-centre audit and review of management

**DOI:** 10.1186/cc12202

**Published:** 2013-03-19

**Authors:** F Gallagher, H Jahn, F Davies

**Affiliations:** 1 Leicester Royal Infirmary, Leicester, UK

## Introduction

Abdominal pain in adolescent females has undergone recent changes with regards to its management under various specialities. The authors report a single-centre audit looking at the correct investigation and management of 12-year-old to 16-year-old girls with abdominal pain in the emergency department setting.

## Methods

A single-centre audit and retrospective analysis of patients took place using case notes and computerised records. Documentation was analysed using statistical analysis and minimum standards were set and reviewed.

## Results

After exclusion criteria 62 females between the ages of 12 and 16 presented to the paediatric emergency department in Leicester with abdominal pain as the predominant admission symptom during a 12-month period. Documentation of the gynaecological history was poor (menstrual history 47%, sexual history 14%, contraception 8%), as was the performance of basic investigations (urine dipsticks 65%, pregnancy test 42%). Documentation was analysed with regard to discharge diagnosis. Ultrasound investigation was performed on seven of the patients but only once admitted to various specialities. No ultrasound was undertaken upon admission.

## Conclusion

Improvement in documentation of minimum standards for these patients is needed. A multidisciplinary care pathway could improve outcome. Consideration should be given to whether early ultrasound investigation is appropriate and there is a further need for investigation as to whether this would improve longer term outcomes.

